# FINDING THE “SO WHAT?”: DEVELOPING A MEANINGFUL CAREER THAT MAKES AN IMPACT

**DOI:** 10.1093/geroni/igad104.1072

**Published:** 2023-12-21

**Authors:** Cal Halvorsen

**Affiliations:** Boston College, Boston, Massachusetts, United States

## Abstract

As a foundation to my social work scholarship, I consistently ask myself a question that my advisor once posed to me: “So what?” In other words, why and to whom does your research matter, and what are you going to do with it? In this presentation, I will show how my scholarship is framed by my practice experience working in Washington, DC for a think tank and advocacy organization. I will describe my approach to merging traditional academic pursuits with public communication and dissemination activities, including publishing in open-access journals, writing op-eds and reports, and pursuing organizational and community collaborations. I will provide specific examples of my collaborative research with local, state, and national organizations, including how we discuss potential research projects and reach mutually beneficial agreements. Lastly, I will describe how I blend this exciting and personally meaningful approach with more established expectations for tenure-track professors.

